# Dietary *cis*-9, *trans*-11-conjugated linoleic acid reduces amyloid β-protein accumulation and upregulates anti-inflammatory cytokines in an Alzheimer’s disease mouse model

**DOI:** 10.1038/s41598-021-88870-9

**Published:** 2021-05-12

**Authors:** Yu Fujita, Kuniyuki Kano, Shigenobu Kishino, Toshihiro Nagao, Xuefeng Shen, Chiharu Sato, Hatsune Hatakeyama, Yume Ota, Sho Niibori, Ayako Nomura, Kota Kikuchi, Wataru Yasuno, Sho Takatori, Kazunori Kikuchi, Yoshitake Sano, Taisuke Tomita, Toshiharu Suzuki, Junken Aoki, Kun Zou, Shunji Natori, Hiroto Komano

**Affiliations:** 1 Division of Neuroscience, School of Pharmacy, Iwate Medical University, 1-1-1 Idaidori, Yahaba-cho, Shiwa-gun, Iwate 028-3694 Japan; 2 Laboratory of Molecular and Cellular Biochemistry, Graduates School of Pharmaceutical Sciences, Tohoku University, Sendai, Miyagi Japan; 3 Division of Applied Life Sciences, Graduate School of Agriculture, Kyoto University, Sakyo-ku, Kyoto Japan; 4 Osaka Research Institute of Industrial Science and Technology, Morinomiya Center, Joto-ku, Osaka Japan; 5 Department of Pharmacy, Japanese Red Cross Morioka Hospital, Morioka, Iwate Japan; 6 Institute for Biomedical Sciences, Library, Iwate Medical University, Nishitokuta, Yahaba-cho, Shiwa-gun, Iwate Japan; 7 Laboratory of Neuropathology and Neuroscience, Graduates School of Pharmaceutical Sciences, Faculty of Pharmaceutical Sciences, The University of Tokyo, Tokyo, Japan; 8 Department of Applied Biological Science, Faculty of Science and Technology, Tokyo University of Science, Noda, Japan; 9 Laboratory of Neuroscience, Graduate School of Pharmaceutical Sciences, Hokkaido University, Sapporo, Japan; 10 Department of Biochemistry, School of Medicine, Nagoya City University, Nagoya, Aichi Japan; 11 Graduates School of Pharmaceutical Sciences, Faculty of Pharmaceutical Sciences, University of Tokyo, Tokyo, Japan; 12 Department of Health Chemistry, Graduate School of Pharmaceutical Sciences, The University of Tokyo, Tokyo, Japan; 13 Present Address: Advanced Prevention and Research Laboratory for Dementia, Graduate School of Pharmaceutical Sciences, Hokkaido University, Sapporo, 060-0812 Japan

**Keywords:** Cell biology, Neuroscience, Diseases, Medical research, Pathogenesis

## Abstract

Conjugated linoleic acid (CLA) is an isomer of linoleic acid (LA). The predominant dietary CLA is *cis*-9, *trans*-11-CLA (*c*-9, *t*-11-CLA), which constitutes up to ~ 90% of total CLA and is thought to be responsible for the positive health benefits associated with CLA. However, the effects of *c*-9, *t*-11-CLA on Alzheimer’s disease (AD) remain to be elucidated. In this study, we investigated the effect of dietary intake of *c*-9, *t*-11-CLA on the pathogenesis of an AD mouse model. We found that *c*-9, *t*-11-CLA diet-fed AD model mice significantly exhibited (1) a decrease in amyloid-β protein (Aβ) levels in the hippocampus, (2) an increase in the number of microglia, and (3) an increase in the number of astrocytes expressing the anti-inflammatory cytokines, interleukin-10 and 19 (IL-10, IL-19), with no change in the total number of astrocytes. In addition, liquid chromatography–tandem mass spectrometry (LC–MS/MS) and gas chromatographic analysis revealed that the levels of lysophosphatidylcholine (LPC) containing *c*-9, *t*-11-CLA (CLA-LPC) and free *c*-9, *t*-11-CLA were significantly increased in the brain of *c*-9, *t*-11-CLA diet-fed mice. Thus, dietary *c*-9, *t*-11-CLA entered the brain and appeared to exhibit beneficial effects on AD, including a decrease in Aβ levels and suppression of inflammation.

## Introduction

Alzheimer’s disease (AD) is a progressive neurodegenerative disorder characterized by loss of memory and cognitive dysfunction, and has risen in prevalence to an estimated 40 million patients worldwide^1,2^. The pathological hallmarks of AD are extracellular senile plaques, which are composed of amyloid-β protein (Aβ), and neurofibrillary tangles^1,2^. Aβ is generated from amyloid precursor protein (APP) through sequential cleavage by two proteases called β- and γ-secretases^1,2^. The two most common isoforms of Aβ are 40 and 42 residues in length, depending on the site of γ-secretase cleavage^1^. Although secreted Aβ40 is much more abundant than Aβ42, Aβ42 is considered to be the causative molecule for triggering the onset of AD because it is more prone to aggregation, more neurotoxic, and the major component in senile plaques^3,4^. Neurofibrillary tangles, which are composed of hyperphosphorylated and aggregated tau in neurons, are another hallmark of AD; however, they are thought to be secondary to amyloid pathology^1,2^. Although many clinical trials have focused on inhibition of Aβ generation, Aβ toxicity, etc., effective treatments to prevent the development of AD have not been firmly established yet^1,2^.

Conjugated linoleic acid (CLA) is an isomer of linoleic acid (LA), which is an essential unsaturated fatty acid^5,6^. CLA is normally generated by symbiotic bacteria in the stomach of ruminant animals^7^. The predominant isomer in dietary sources is *cis*-9, *trans*-11-CLA (*c*-9, *t*-11-CLA), which constitutes up to ~ 90% of total CLA, and is present at relatively higher levels in the meat and milk fat of ruminant animals^6,7^. *Trans*-10, *cis*-12-CLA (*t*-10, *c*-12-CLA) is another common isomer that accounts for 1–10% of total CLA in dietary sources^6,7^. Recent studies using animal models or cultured cells have reported that CLA has beneficial effects on health, including effects on atherosclerosis^8,9^, colitis^10^, metabolic syndrome^9^, rheumatoid arthritis^11^, carcinogenesis ^9,12^, and immune cell function^13,14^. However, most of the published studies used a mixture of the two major CLA isomers, and which isomer is responsible for these functions is not clear. Accumulating evidence demonstrates that *c*-9, *t*-11-CLA has benefits associated with CLA, whereas *t*-10, *c*-12-CLA is associated with the anti-obesity effects seen with CLA^5,6^. In addition, in vitro studies using *c*-9, *t*-11-CLA have shown several biological effects on neurons, including promotion of proliferation of neuronal progenitor cells^15^ and protection from glutamate-induced or Aβ-induced neuronal cell death^16,17^, suggesting beneficial effects of *c*-9, *t*-11-CLA on neurodegenerative disorders including AD. However, the effects of CLA on AD remain unknown.

Therefore, in this study, we focused on *c*-9, *t*-11-CLA and investigated the effects of a *c*-9, *t*-11-CLA diet on AD pathology using AD model mice. Our results indicated that dietary *c*-9, *t*-11-CLA indeed entered the brain and exhibited beneficial effects on AD, including a decrease in Aβ accumulation and an enhanced anti-inflammatory effect.

## Results

### A *c*-9, *t*-11-CLA diet influenced the pathogenesis of AD model mice, including Aβ accumulation

**Figure 1:**
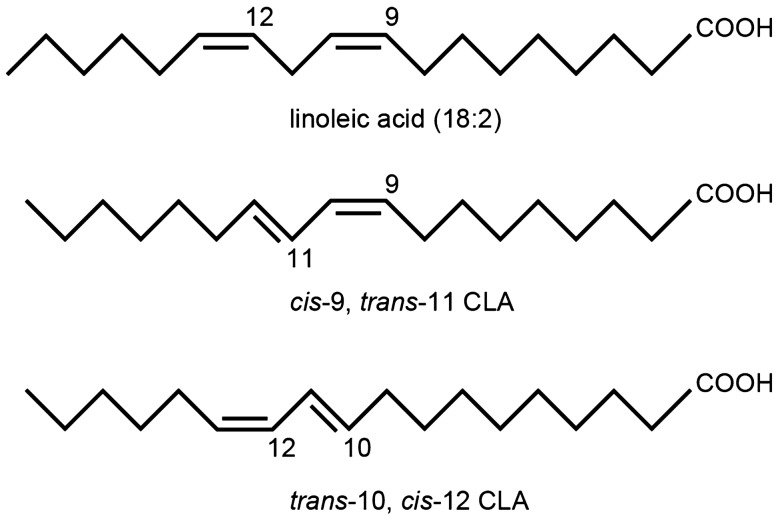
Structure of linoleic acid and its two main conjugated derivatives. CLA is normally generated by symbiotic bacteria in the stomach of ruminant animals^7^. The predominant isomer in dietary sources is *cis*,*trans*-11-CLA ( c-9, t-11-CLA) (second panel), which constitutes up to ~ 90% of total CLA^6,7^ Trans-10, cis-12-CLA (t-10, c-12-CLA) (bottom panel) is another common isomer that accounts for 1–10% of total CLA in dietary sources^6,7^.

Because *c*-9, *t*-11-CLA is likely to be responsible for the positive health benefits associated with CLA^5^, we focused on *c*-9, *t*-11-CLA instead of *t*-10, *c*-12-CLA on AD pathology (Fig. 1).

**Figure 2:**
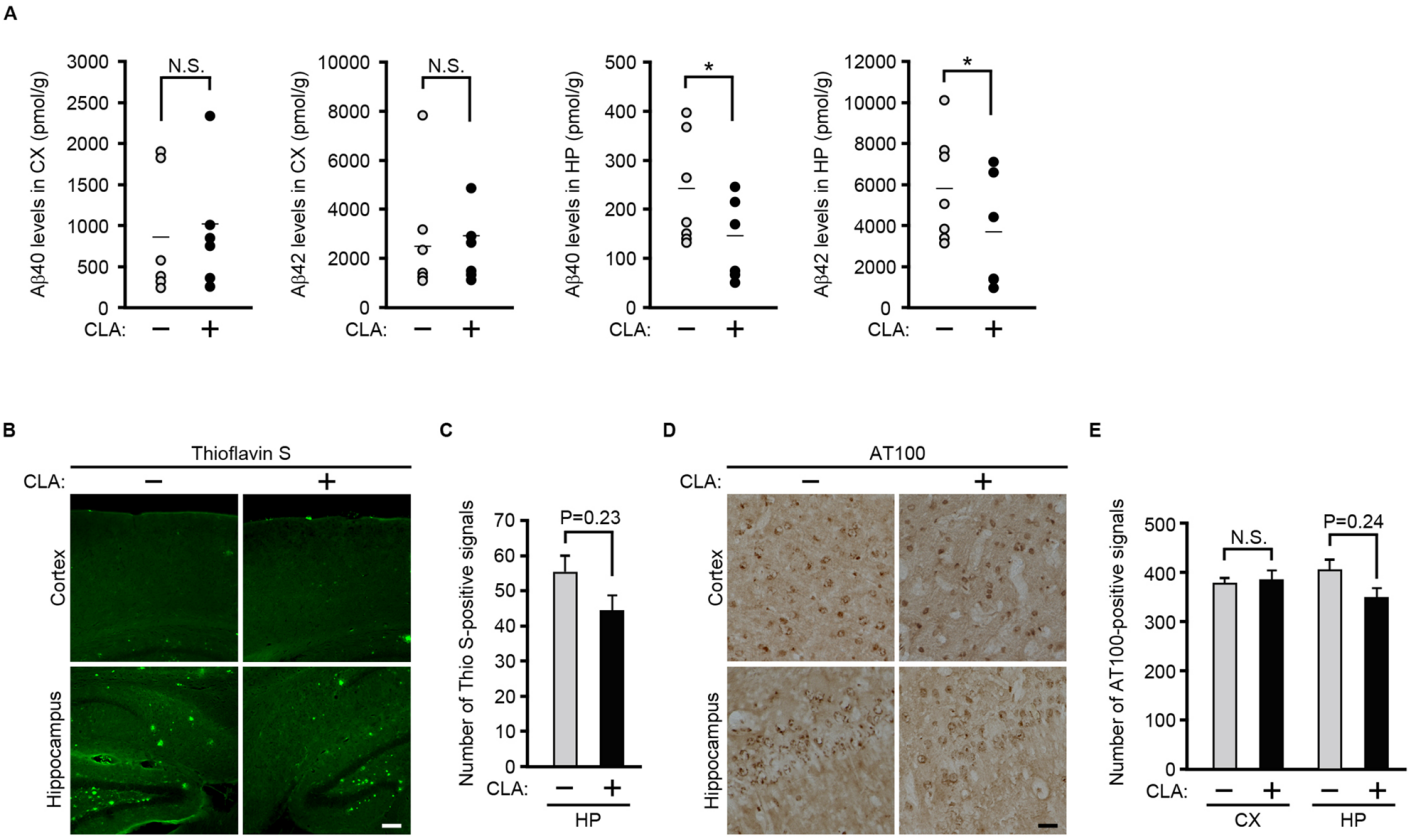
The *c*-9, *t*-11-CLA diet reduces Aβ40 and Aβ42 levels in the hippocampus of AD model mice. (**A**) Aβ40 and Aβ42 levels were measured with ELISA using lysates of the cortex and hippocampus of *c*-9, *t*-11-CLA diet-fed AD model mice (CLA +) and control diet-fed AD model mice (CLA −). Aβ40 and Aβ42 levels in the hippocampus of *c*-9, *t*-11-CLA diet-fed mice were significantly decreased (right graphs) compared with controls, but were not significantly changed in the cortex (left graphs). Bars show the average value (n = 6 mice for each group). *P < 0.05 (Aβ40, *P* = 0.032; Aβ42, *P* = 0.031). (**B**) Thioflavin-S staining of brain sections of *c*-9, *t*-11-CLA diet-fed and control diet-fed AD model mice. The number of thioflavin-S-positive signals (green) tended to decrease in the hippocampus, but the positive signals were rarely detected in the cortex. (C) The histograms show the number of thioflavin-S-positive signals in the hippocampus. (n = 6 mice for each group; five different fields per mouse were used for counting of the positive signals). (**D**) Immunostaining of the brain sections of *c*-9, *t*-11-CLA diet-fed and control diet-fed AD model mice with AT100 antibody. The number of AT100-positive signals in the hippocampus of *c*-9, *t*-11-CLA diet-fed mice tended to decrease but no difference was seen in the cortex. (**E**) The histograms show the number of AT100-positive signals (n = 6 mice for each group; eight fields per mouse were counted). CX: cortex, HP: hippocampus. Bar, 50 µm. CLA + : *c*-9, *t*-11-CLA diet-fed AD model mice. CLA − : control diet-fed AD model mice.

Aβ is thought to be a major pathogenesis factor in AD. Therefore, we first investigated whether dietary *c*-9, *t*-11-CLA supplementation affects Aβ accumulation in the brain of AD model mice. For this purpose, we measured Aβ levels in the brain using an enzyme-linked immunosorbent assay (ELISA). We found that Aβ42 and Aβ40 levels in the hippocampus of *c*-9, *t*-11-CLA diet-fed AD model mice were significantly lower than those of control mice, but levels were not significantly different in the cortex (Fig. 2A). We further conducted thioflavin-S staining to detect Aβ deposition in the brain. As shown in Fig. 2B, C, the number of thioflavin-S-positive plaques tended to decrease in the hippocampus in *c*-9, *t*-11-CLA diet-fed AD model mice, although thioflavin-S-positive plaques were rarely detected in the cortex. Because hyperphosphorylated tau is another hallmark of AD, we next performed immunostaining with anti-AT100 antibody (anti-phosphorylated tau antibody). As shown in Fig. 2D, E, AT100-positive signals also tended to decrease in the hippocampus of *c*-9, *t*-11-CLA diet-fed AD model mice, but they did not change in the cortex. Taken together, feeding the *c*-9, *t*-11-CLA diet reduced the Aβ level in the hippocampus of AD model mice, and this reduction is likely to decrease Aβ deposition and tau phosphorylation.

### The ***c***-9, ***t***-11-CLA diet increased the number of microglia, including CD45^+^ and CD206^+^ microglia, in the cortex and hippocampus of AD model mice, but not the number of astrocytes

**Figure 3:**
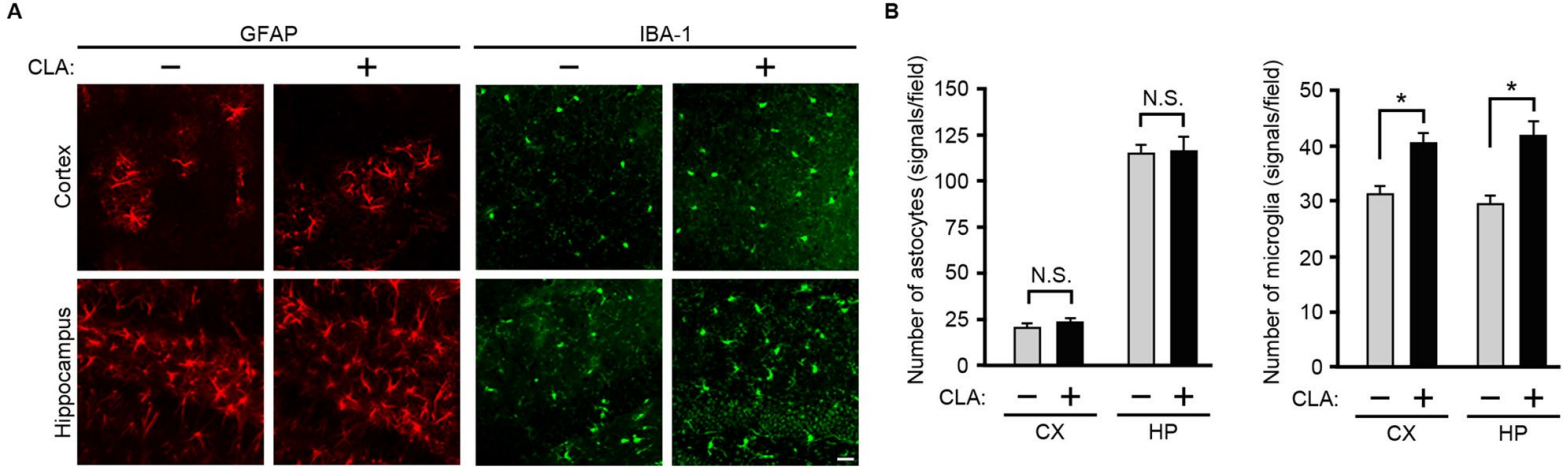
Effect of the *c*-9, *t*-11-CLA diet on the number of microglia and astrocytes in the brain of AD model mice. (**A**) Immunostaining of the brain sections of *c*-9, *t*-11-CLA diet-fed and control diet-fed AD model mice with anti-GFAP (astrocyte marker) and anti-IBA-1 antibodies (microglia marker). The number of IBA-1 (green)-positive signals was significantly increased in the cortex and hippocampus of *c*-9, *t*-11-CLA diet-fed mice compared with the control (right panels), whereas the number of GFAP (red)-positive signals was not changed (left panels). (**B**) The histograms show the numbers of GFAP-positive and IBA-1-positive signals (n = 6 mice for each group; five different fields per mouse were used to count the number). GFAP: glial fibrillary acidic protein; IBA-1: ionized calcium binding adaptor molecule 1. Bar, 20 µm. **P < 0.01 (cortex, *P* = 7.3 × 10^−8^; hippocampus, *P* = 3.8 × 10^−5^).

Astrocytes and microglia are thought to be involved in Aβ clearance in the brain^18,19^. Therefore, we next investigated whether feeding the *c*-9, *t*-11-CLA diet affects the number of astrocytes and microglia in the brain of AD model mice. We conducted immunostaining of brain sections with anti-glial fibrillary acidic protein (GFAP) (astrocyte marker) and anti-ionized calcium binding adaptor molecule 1 (IBA-1) (microglia marker) antibodies. We found that IBA-1-positive signals were significantly increased in the cortex and hippocampus of *c*-9, *t*-11-CLA diet-fed AD model mice, but GFAP-positive signals did not change (Fig. 3A, B). These results suggest that the *c*-9, *t*-11-CLA diet increased the number of microglia in the brain of AD model mice but did not affect the number of astrocytes.

We next determined the subpopulations of microglia that are increased by a *c*-9, *t*-11-CLA-diet, because microglia are not homogeneous and several subpopulations have been identified as disease-associated microglia (DAM)^20^, or inflammatory or anti-inflammatory phenotypes^21^. For this purpose, we performed immunostaining of microglia with antibodies for CD45 (a transmembrane protein tyrosine phosphatase for a microglia subtype), CD86 (surface antigen cluster of differentiation 86 for M1 marker activated by pathogens or pro-inflammatory factors), and CD206 (a mannose receptor for M2 marker activated by anti-inflammatory factors such as IL-10)^21^.

**Figure 4:**
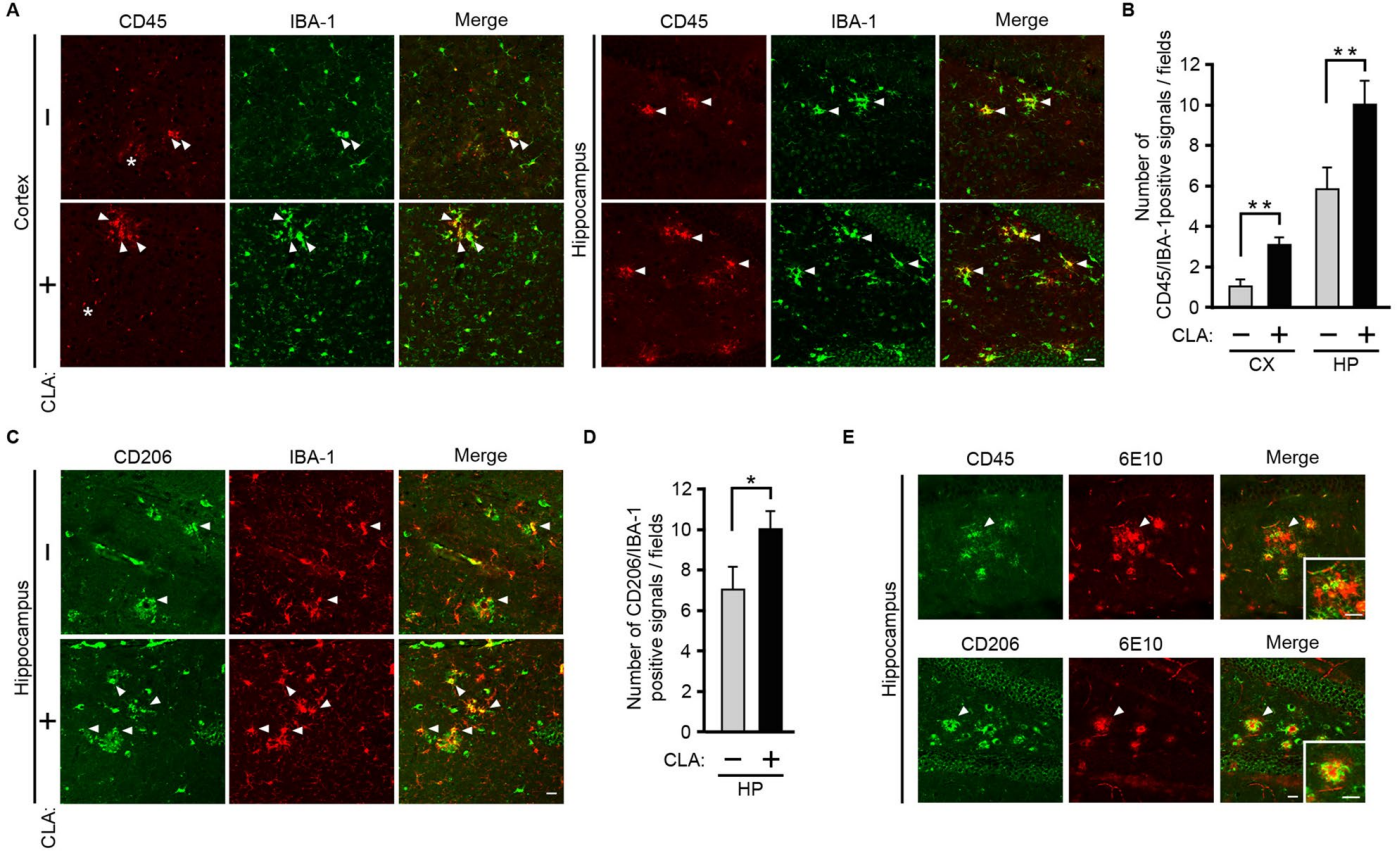
The *c*-9, *t*-11-CLA diet increased the number of CD45^+^ and CD206^+^ microglia. (**A**) Double immunostaining of brain sections of *c*-9, *t*-11-CLA diet-fed and control diet-fed AD model mice with anti-CD45 and anti-IBA-1 (microglia marker) antibodies. A portion of IBA-1 (green)-positive cells were CD45 (red)-positive as shown by the yellow color (right merged panels). The arrows indicate the representative CD45 (red)-and IBA-1 (green)-positive cells as shown by yellow color (right merged panels). Small red dots (representatives marked with an asterisk) were nonspecifically stained as judged from the staining of the brain sections without the 1^st^ antibody. Bar, 20 µm. WT: wild-type mice, AD: AD model mice. CLA + : *c*-9, *t*-11-CLA diet-fed AD mouse model; CLA − : control diet-fed AD model mice. (**B**) Histograms show the numbers of CD45-positive and IBA-1-positive signals (n = 6 mice for each group; five different fields per mouse were counted). **P < 0.01 (hippocampus, *P* = 0.01; cortex, *P* = 0.0097). (**C**) Double immunostaining of brain sections in hippocampus of *c*-9, *t*-11-CLA diet-fed and control diet-fed AD model mice with anti-CD206 and anti-IBA-1 (microglia marker) antibodies. The arrows indicate the representative CD206 (red)-and IBA-1 (green)-positive cells as shown by yellow color (right merged panels). Brain sections in the cortex was not clearly immunostained with anti-CD206 antibody (data not shown). (**D**) Histograms show the numbers of CD206-positive and IBA-1-positive signals (n = 6 mice for each group; five different fields per mouse were counted). (**E**) CD45^+^ and CD206^+^ microglia were co-localized with Aβ deposits. The brain sections in hippocampus of *c*-9, *t*-11-CLA diet-fed AD model mice were doubly immunostained with anti-CD45 (green) and anti-Aβ (red) antibodies (upper panels) or anti-CD206 and anti-Aβ (red) antibodies (lower panels). The arrow indicates the representative CD45 (green)- or CD206- and 6E10 (red)-positive cells as shown by yellow color (right merged panels). 6E10 was used for the anti-Aβ (red) antibody. The insets in right panels show the magnified pictures indicated by arrows. Bar, 20 µm.

We first found that CD45-positive (CD45^+^) cells were only observed in AD model mice (supplemental Fig. 1), which is consistent with the notion that CD45^+^ microglia are associated with AD pathology^20^. We also confirmed that CD45^+^ cells are all stained with anti-IBA (microglia marker) antibodies, showing that CD45^+^ cells are microglia (as shown in Fig. 4A). Therefore, we next determined with double staining of anti-CD45 and anti-IBA antibodies whether CD45^+^ microglia are increased by the *c*-9, *t*-11-CLA diet. The results showed that CD45- and IBA-positive microglia were increased by *c*-9, *t*-11-CLA feeding (Fig. 4A, B). However, CD86-positive cells (for the M1 phenotype marker) were not found in either *c*-9, *t*-11-CLA-fed or control-fed mouse brains (data not shown). We also found that CD206- and IBA-positive microglia were increased in *c*-9, *t*-11-CLA-fed AD mice (Fig. 4C, D). We also noted that CD45^+^ microglia and CD206^+^ microglia existed in Aβ deposits (Fig. 4E). CD45 is associated with AD pathology and appears to play a role in the clearance of oligomeric Aβ^22^, and CD206^+^ microglia are likely to be involved in anti-inflammation^21^.

### The number of astrocytes expressing interleukin (IL)-10 or IL-19 was increased in the hippocampus of *c*-9, *t*-11-CLA diet-fed AD model mice

**Figure 5:**
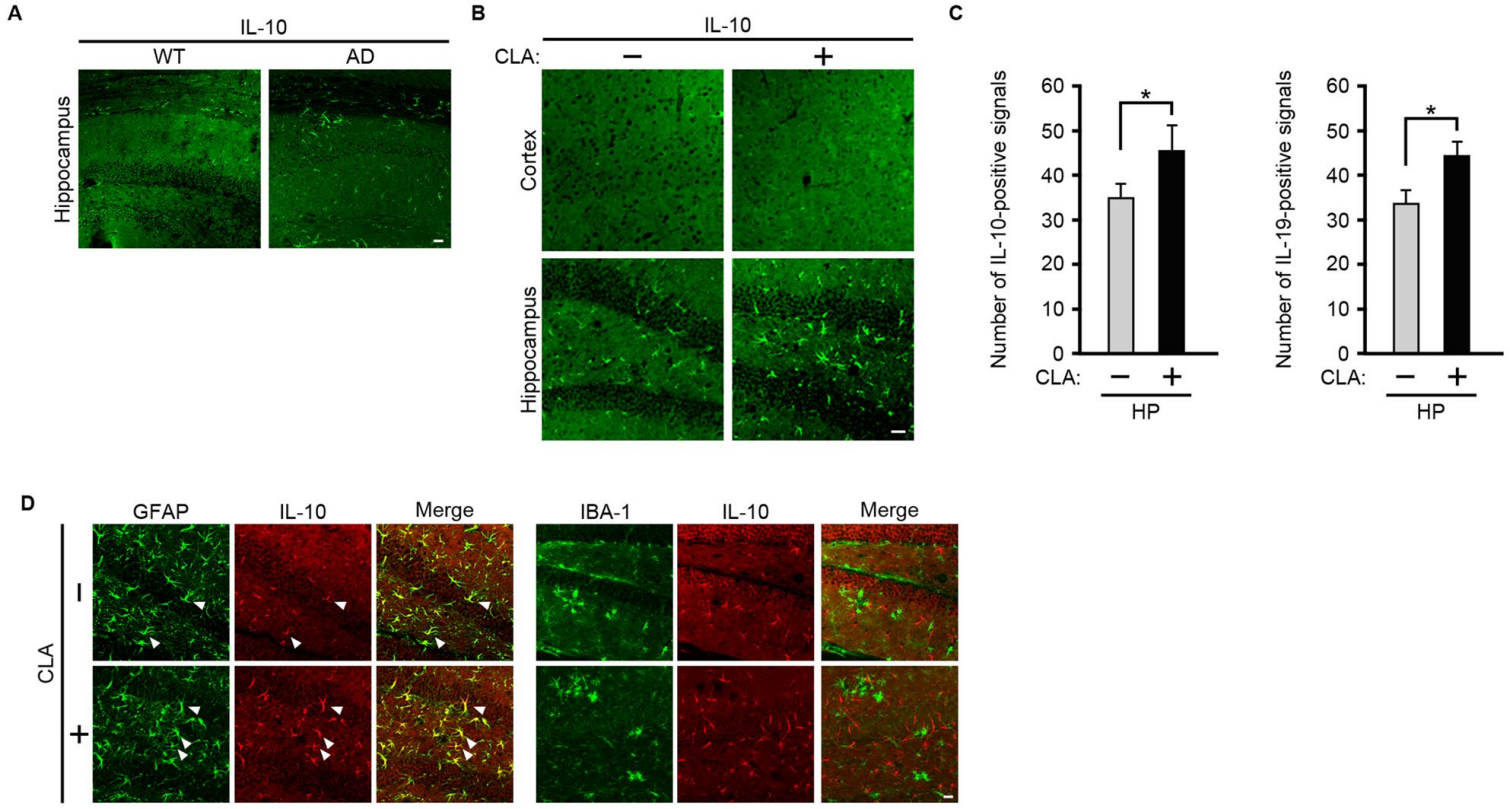
The *c*-9, *t*-11-CLA diet upregulates the number of IL-10- or IL-19-expressing astrocytes in the hippocampus of AD model mice. (**A**) Immunostaining of brain sections of wild-type and AD model mice with anti-IL-10 antibody. (**B**) Immunostaining of *c*-9, *t*-11-CLA diet-fed and control diet-fed AD model mice with anti-IL-10 antibody. IL-10 (green)-positive cells were significantly increased in the hippocampus of *c*-9, *t*-11-CLA diet-fed AD model mice compared with controls (lower panels). IL-10 (green)-positive cells were not observed in the cortex (upper panels). (**C**) The histogram shows the number of IL-10-positive cells (left panel) and IL-19-positive cells (right panel) in the hippocampus. (n = 6 mice for each group; three different fields per mouse were used to count the number). *P < 0.05 (IL-10, *P* = 0.023; IL-19, *P* = 0.04). HP: hippocampus (**D**) Double immunostaining of brain sections in the hippocampus of *c*-9, *t*-11-CLA diet-fed and control diet-fed AD model mice with anti-GFAP (astrocyte marker), anti-IBA-1 (microglia marker), and anti-IL-10 antibodies. A portion of GFAP (green)-positive cells were IL-10 (red) positive as shown by yellow color (left merge panels), but IBA-1 (green)-positive cells were not co-localized with IL-10 (red)-positive cells (right merge panels). The arrows indicate the representative GFAP (green)- and IL-10 (red)-positive cells as shown by yellow color (right merged panels). Bar, 20 µm. WT: wild-type mice, AD: AD model mice. CLA + : *c*-9, *t*-11-CLA diet-fed AD mouse model, CLA − : control diet-fed AD model mice.

As *c*-9, *t*-11-CLA treatment was previously shown to enhance the expression levels of the anti-inflammatory cytokine, IL-10^23^, we next examined whether the *c*-9, *t*-11-CLA diet affects the expression of IL-10 in the brain. We first performed immunostaining of wild-type and AD mouse model brain sections with an anti-IL-10 antibody. IL-10-positive signals were clearly detected in the hippocampus of the brain of AD model mice, but not in wild-type mouse brain (Fig. 5A), indicating that IL-10 expression was upregulated in the brain of AD model mice. We then compared the number of IL-10-positive signals in the brain of *c*-9, *t*-11-CLA diet-fed and control diet-fed AD model mice. The number of IL-10-positive signals was significantly increased in the hippocampus of *c*-9, *t*-11-CLA diet-fed AD model mice, although they were rarely detected in the cortex (Fig. 5B, C). Because IL-10 is expressed in astrocytes and microglia in the brain^24^, we next performed double immunostaining with anti-IL-10 antibodies and anti-GFAP and anti-IBA-1 to determine which cell type, astrocytes or microglia, showed upregulated IL-10 expression. As shown in Fig. 5D, some GFAP-positive signals in the hippocampus clearly overlapped with IL-10-positive signals, but IBA-1-positive signals did not overlap with IL-10. These results indicate that IL-10 was expressed in a portion of astrocytes, but not in microglia, and that the *c*-9, *t*-11-CLA diet increased the number of astrocytes expressing IL-10. We also found that the *c*-9, *t*-11-CLA diet increased the number of astrocytes expressing IL-19, which is another anti-inflammatory cytokine^24^ (Fig. 5C and supplemental Fig. 2). In addition, we attempted to immunodetect other inflammatory cytokines such as IL-1β, IL-6, and tumor necrosis factor (TNF)α, but they were all below the level of detection in the AD model mice used in our study (data not shown).

### The *c*-9, *t*-11-CLA diet increased the levels of *c*-9, *t*-11-CLA-lysophosphatidylcholine (LPC) and free *c*-9, *t*-11-CLA in the brain

**Figure 6:**
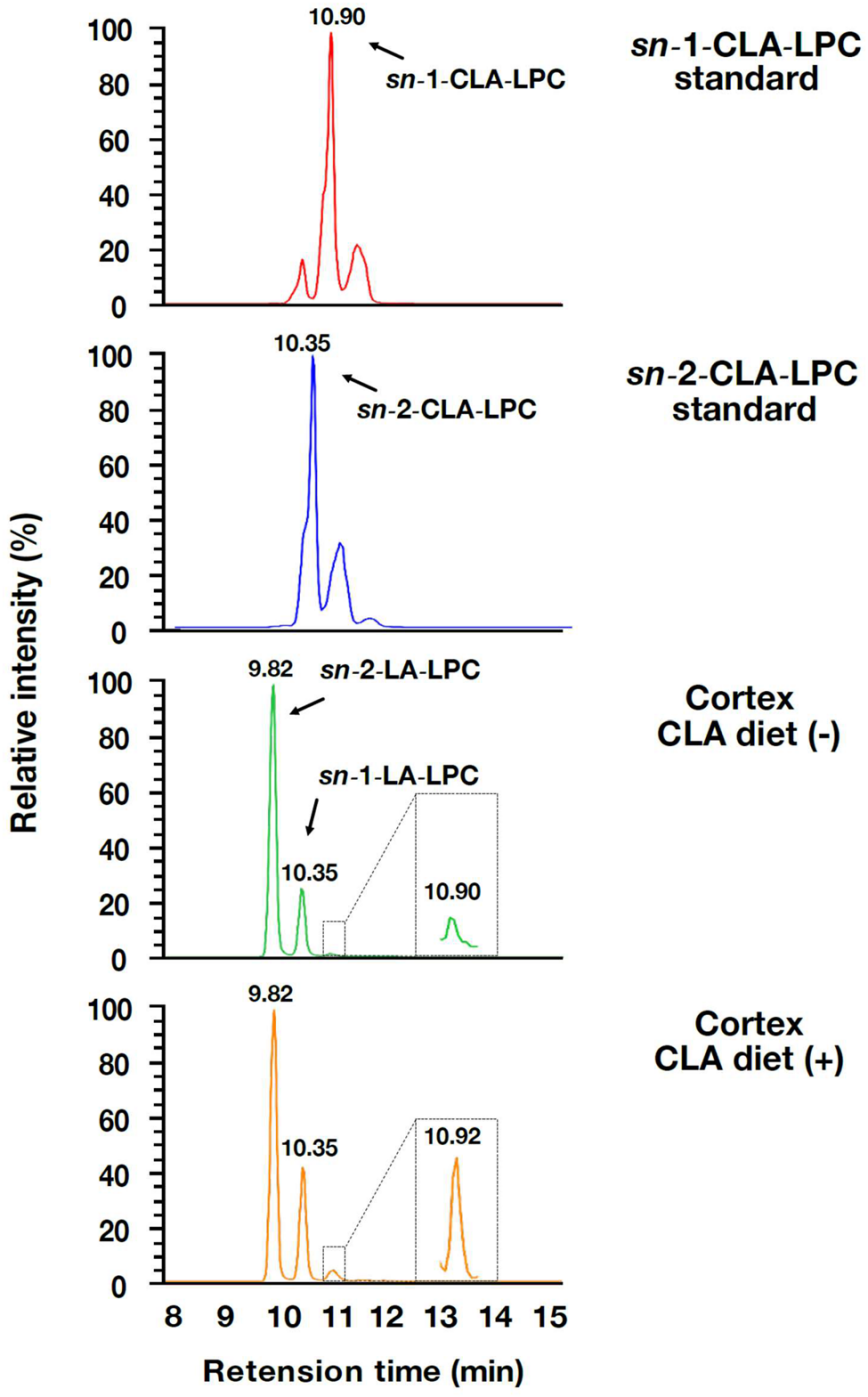
LC–MS/MS analysis of synthetic *sn*-1- and *sn*-2-CLA-LPC. Synthetic *sn*-1-CLA-LPC (top panel), synthetic *sn*-2-CLA-LPC (second panel from the top), the LPC peak area of the cortex lysate from control diet-fed mice (third panel from the top), and the LPC peak area of the cerebellum lysate from CLA diet-fed mice (bottom panel) were detected with LC–MS/MS analysis. Synthetic *sn*-1-CLA-LPC corresponded to the extra peak with a delayed retention time following the two peaks of LA-LPC (*sn*-1-LA and *sn*-2-LA. See^27^) observed in the cortex, whereas the peak of *sn*-2-CLA-LPC corresponded to that of *sn*-1-LA-LPC.

**Figure 7:**
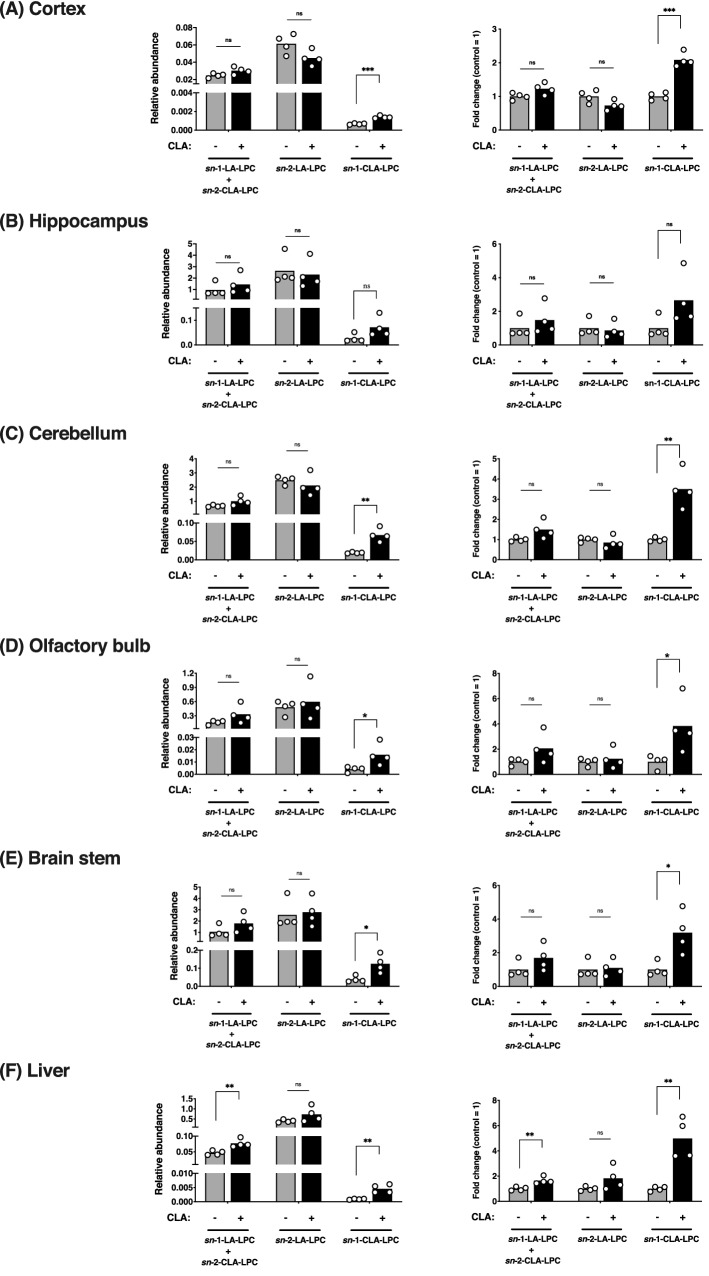
The *c*-9, *t*-11-CLA-LPC level in the brain was increased by the *c*-9, *t*-11-CLA diet. The LPC peaks in the various brain regions and the liver were detected with LC–MS/MS analysis. Relative abundance is shown as the area ratio per tissue weight (g) (left panel), and the fold change was indicated as the relative ratio of the peak area in *c*-9, *t*-11-CLA diet-fed mice to that in control diet-fed mice (right panel). The peak of *sn*-1-*c*-9, *t*-11-CLA-LPC in the lysates of the cortex (*P* = 0.00068) (**A**), cerebellum (*P* = 0.0017) (**C**), olfactory bulb (*P* = 0.04) (**D**) and brain stem (*P* = 0.015) (**E**) was significantly increased in *c*-9, *t*-11-CLA diet-fed mice as well as in the liver (*P* = 0.0025) (**F**). The peak for *sn*-1-*c*-9, *t*-11-CLA-LPC in the hippocampus (*P* = 0.09) was not significantly changed, but it tended to be increased. A peak with no significant change in area for LA [*sn*-1-(and also *sn*-2-CLA-) and *sn*-2-LA]-LPC was observed between brain regions from *c*-9, *t*-11-CLA diet-fed and control diet-fed mice, but an increased tendency by the *c*-9, *t*-11-CLA diet was observed. In the liver, the peak of *sn*-1-LA-LPC and also *sn*-2-CLA-LPC was significantly increased by the *c*-9, *t*-11-CLA diet. Left panel: LPC area ratio/g tissue. n = 4 mice for each group. *P < 0.05, **P < 0.001, ***P < 0.0001 ns: not significant.

We finally determined whether dietary *c*-9, *t*-11-CLA entered the brain. Plasma fatty acids, especially docosahexaenoic acid, are transported into the brain in the form of LPC by the Mfsd2a transporter^25,26^. Therefore, we first examined the level of LPC containing *c*-9, *t*-11-CLA (*c*-9, *t*-11-CLA-LPC) in the brain. To detect and quantify *c*-9, *t*-11-CLA-LPC, we used a reverse-phase liquid chromatography (LC) method for separating the LPC structural isomer, LA-LPC, and *c*-9, *t*-11-CLA-LPC. To identify the peak chromatogram of *c*-9, *t*-11-CLA-LPC, we performed LC–MS/MS analysis of synthetic *c*-9, *t*-11-CLA-LPC. As shown in Fig. 6, synthetic *sn*-1-CLA-LPC corresponded completely to the extra peak with a delayed retention time following the two peaks of LA-LPC (*sn*-1-LA and *sn*-2-LA. See^27^), which was previously suggested to be that of *c*-9, *t*-11-CLA-LPC (Hata et al., submitted elsewhere; https://biorxiv.org/cgi/content/short/2020.09.13.295642v1), whereas the peak of *sn*-2-CLA-LPC corresponded to that of *sn*-1-LA-LPC. We then determined whether the peaks of CLA-LPC were increased in the brain of *c*-9, *t*-11-CLA diet-fed mice. As shown in Fig. 7, we found that the *sn*-1-CLA-LPC level was increased by approximately twofold in the brain of *c*-9, *t*-11-CLA diet-fed mice, including the cortex (*P* = 0.0006), hippocampus (*P* = 0.09), cerebellum (*P* = 0.0017), brain stem (*P* = 0.015), and olfactory bulb (*P* = 0.04), as well as in the liver (*P* = 0.0025), whereas no change in *sn*-2-LPC-LA was observed with *c*-9, *t*-11-CLA feeding. We also noted that the relative abundance of *sn*-1-CLA-LPC in the hippocampus and brain stem was much higher than that in the other brain regions, including the cortex, and in the liver (Fig. 7, left panels).

However, a significant increase in the level of *sn*-1-LA-LPC including *sn*-2-CLA-LPC, in *c*-9, *t*-11-CLA diet-fed mice was not observed except for in the liver, although the level tended to be increased. Other lysophospholipids containing *c*-9, *t*-11-CLA, including lysophosphatidylserine etc., were below the level of detection. Therefore, dietary *c*-9, *t*-11-CLA appeared to form *c*-9, *t*-11-CLA-LPC and then enter the brain.

**Figure 8:**
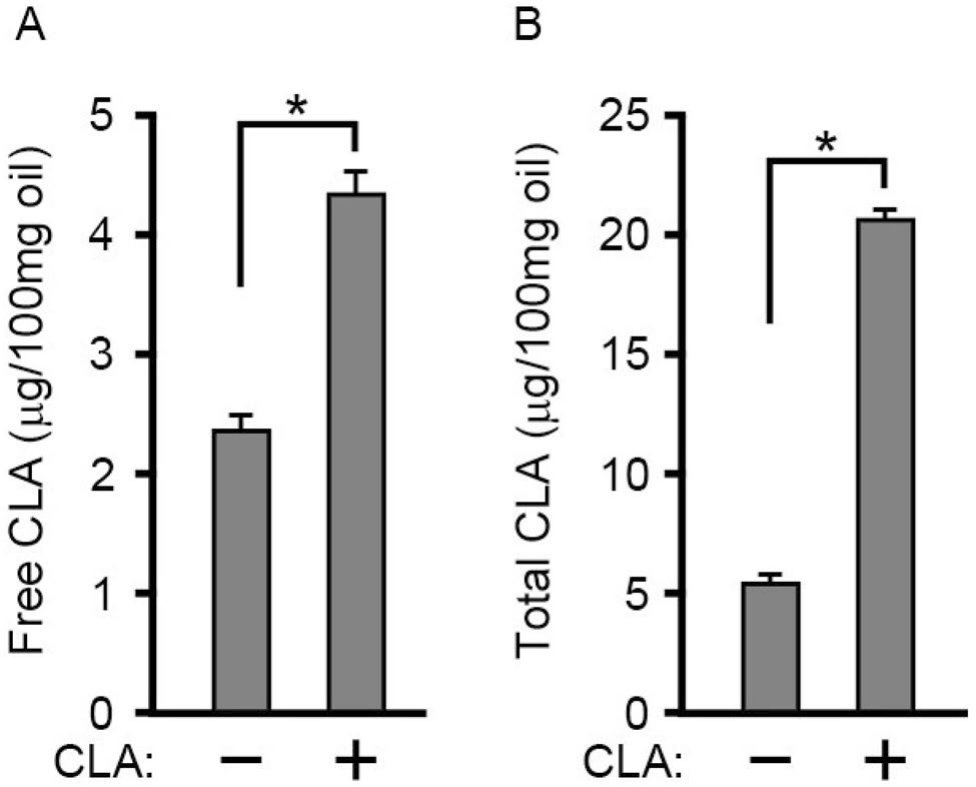
Gas chromatographic analysis of the level of free *c*-9, *t*-11-CLA and total *c*-9, *t*-11-CLA in the brain of *c*-9, *t*-11-CLA diet-fed mice. Free *c*-9, *t*-11-CLA was quantitatively determined in the brains from control diet-fed mice ( −) and *c*-9, *t*-11-CLA diet-fed mice as described in “Methods”. (**B**) Total *c*-9, *t*-11-CLA was quantitatively determined by gas chromatography after saponification of the extracted lipids from the brains as described in “Methods.” *P < 0.05 (free CLA, *P* = 0.0002; total CLA, *P* = 1.6 × 10^−6^).

We then performed gas chromatographic analysis to determine whether the level of free *c*-9, *t*-11-CLA was increased in *c*-9, *t*-11-CLA diet-fed mice. As shown in Fig. 8, the level of free *c*-9, *t*-11-CLA was significantly increased by approximately twofold, and the total level of *c*-9, *t*-11-CLA was also increased by approximately fourfold in *c*-9, *t*-11-CLA diet-fed mice. Thus, dietary *c*-9, *t*-11-CLA clearly entered the brain, possibly mediated by *c*-9, *t*-11-CLA-LPC, thereby increasing the level of free *c*-9, *t*-11-CLA in the brain.

## Discussion

In the present study, we determined the effect of dietary *c*-9, *t*-11-CLA on the pathogenesis of AD using AD model mice.

The major dietary sources of *c*-9, *t*-11-CLA are dairy products and ruminant meat, whereas those of *t*-10, *c*-12-CLA are partially hydrogenated vegetable oils from margarines and shortenings^5,6^. A growing number of studies has demonstrated that *c*-9, *t*-11-CLA is responsible for the positive health benefits associated with CLA, whereas *t*-10, *c*-12-CLA is associated with the anti-obesity effects seen with CLA^5^. However, the effect of dietary CLA on AD pathogenesis is not known. Therefore, we determined the effect of a *c*-9, *t*-11-CLA diet on AD pathology.

We first found that dietary intake of *c*-9, *t*-11-CLA reduced Aβ42 and Aβ40 accumulation in the hippocampus. However, Aβ deposits and tau phosphorylation tended to decrease, but did not significantly change. For the formation of Aβ deposits or tau phosphorylation, the ratio of Aβ42 versus Aβ40 is thought to be crucial, because Aβ42 is much more prone to aggregation than Aβ40 and thereby forms the seed of Aβ deposition^3–5^. The decreases in Aβ deposition and tau phosphorylation were not drastic despite the decrease in the Aβ level by *c*-9, *t*-11-CLA feeding, probably because the Aβ level is decreased without changing the ratio of Aβ42 versus Aβ40.

Interestingly, we found that the decrease in Aβ accumulation was accompanied by upregulation of the anti-inflammatory cytokines, IL-10 and IL-19. We also found that *c*-9, *t*-11 CLA feeding increased the number of microglia in both the cortex and hippocampus. In general, microglia are involved in the elimination of unnecessary substances such as amyloid plaques and apoptotic cells in the brain^2,19,22^. Therefore, our results suggest that increasing the number of microglia by *c*-9, *t*-11-CLA may promote Aβ clearance. In fact, we found that CD45^+^ microglia, which could be involved in Aβ clearance^22^, were increased by *c*-9, *t*-11 CLA feeding as will be described later, strongly suggesting that *c*-9, *t*-11-CLA may promote microglia-mediated Aβ clearance. We also demonstrated that astrocytes expressing IL-10 were significantly increased in the hippocampus following *c*-9, *t*-11-CLA feeding. Astrocytes are also involved in Aβ clearance, including engulfment of Aβ and secretion of the protease for Aβ degradation^28,29^. Although it is not exactly known why the levels of Aβ40 and Aβ42 in the cortex were not significantly decreased in *c*-9, *t*-11-CLA diet-fed AD model mice, we assumed that astrocytes expressing IL-10 either alone or together with increased microglia could be involved in reducing the Aβ level in their hippocampi. As the relative abundance of *c*-9, *t*-11-CLA-LPC in the hippocampus was approximately 100-fold higher than that in the cortex (Fig. 7, left panel), this may be related to the stronger effects of *c*-9, *t*-11-CLA feeding on AD pathology in the hippocampus than in the cortex.

Because we also showed that treatment with *c*-9, *t*-11-CLA reduces Aβ production in vitro (Hata et al., submitted elsewhere; https://biorxiv.org/cgi/content/short/2020.09.13.295642v1), another possibility is that *c*-9, *t*-11-CLA directly decreased Aβ generation in neurons. There appear to be several mechanisms underlying the reduction of Aβ levels by the *c*-9, *t*-11-CLA diet, including a reduction in Aβ generation and the promotion of Aβ clearance.

We also found that CD45^+^ microglia and CD206^+^ microglia were increased by *c*-9, *t*-11-CLA feeding, while CD86-positive cells were not found in either *c*-9, *t*-11-CLA-fed or control-fed mouse brains. CD45^+^ microglia are thought to be DAM and have been found to be associated with AD. A study using CD45-deficient mice has demonstrated CD45-mediated microglial clearance of oligomeric Aβ^22^. Therefore, the reduction in Aβ levels by *c*-9, *t*-11-CLA feeding may occur by promoting differentiation into CD45^+^ microglia. We also found that CD206^+^ microglia, which appear to play a role in anti-inflammation, were increased by *c*-9, *t*-11-CLA. CD206^+^ microglia are thought to have the M2 phenotype, which can shut down ongoing inflammation and promote recovery^21^. M2 activation is induced by the presence of anti-inflammatory cytokines such as IL-10. We assume that an increase in the number of astrocytes expressing IL-10 or IL-19 by *c*-9, *t*-11-CLA feeding may lead to the induction of CD206^+^ microglia. However, CD86-positive microglia, which are activated by inflammation and exhibit the M1 phenotype for inflammation, were not found in our study (data not shown).

At present, it is not known whether *c*-9, *t*-11-CLA feeding increased microglia derived from brain cells or from peripheral immune cells, because it is still controversial whether DAM are all derived from brain microglia or peripheral immune cells^20^. However, a recent study strongly suggested that DAM all come from brain microglia^30^. Further studies will be needed to see how *c*-9, *t*-11-CLA leads to an increase in the number of some subpopulations of microglia.

Our results also showed that the number of IL-10- or IL-19-expressing cells increased in the hippocampus of *c*-9, *t*-11-CLA-fed AD model mice. IL-10 and IL-19 were expressed in astrocytes but not in microglia in our AD model mice. Astrocytes have neuroprotective functions by secreting anti-inflammatory cytokines, such as IL-10, IL-19, and transforming growth factor-β, and repairing the blood–brain barrier (BBB) to restrict the migration of leukocytes caused by the inflammation^12^. Thus, our results suggest that *c*-9, *t*-11-CLA feeding increases the number of IL-10- or IL-19-expressing astrocytes, resulting in suppression of inflammation in the AD brain. We also attempted to detect inflammatory cytokines such as IL-1β, IL-6, and TNFα in the brain, but they were all below the level of detection in the AD model mice used in this study (data not shown), which appears to be consistent with the lack of inflammatory CD86-positive microglia. As IL-10 is induced even in control diet-fed AD model mice, upregulated IL-10 or IL-19 could suppress the expression of inflammatory cytokines such as IL-1β, IL-6, and TNF. As previously reported, the treatment of dendritic cells with *c*-9, *t*-11-CLA induces IL-10 production in vitro^23^, and we also examined whether treatment of primary glial cells and glial cell lines with *c*-9, *t*-11-CLA induces IL-10 production. However, we failed to detect an increase in the levels of IL-10 in primary cultured glial cells by *c*-9, *t*-11-CLA treatment (data not shown). Therefore, the upregulation of IL-10 in astrocytes in the brain of *c*-9, *t*-11-CLA diet-fed mice appears to be due to secondary effects of *c*-9, *t*-11-CLA or to metabolites derived from *c*-9, *t*-11-CLA, such as lysophospholipids or diacyl phospholipids, as described below. *c*-9, *t*-11-CLA can activate, one of the nuclear transcription factors, peroxisome proliferator-activated receptor gamma (PPARγ), the ligand-dependent activation of which dramatically inhibits the cellular immune response and production of inflammatory mediators^31^. Therefore, dietary intake of *c*-9, *t*-11-CLA might only regulate the inflammatory response, although at present it is not known whether it also affects the functions of microglia and astrocytes in wild-type mice.

The neuroinflammation caused by Aβ accumulation is thought to be crucial for the loss of memory in AD^32^. In this regard, dietary *c*-9, *t*-11-CLA supplementation will be useful for preventing AD progression, because dietary *c*-9, *t*-11-CLA upregulated anti-inflammatory cytokines in the brain, and also possibly inhibited neuroinflammation through the activation of PPARγ. We also note that dietary *c*-9, *t*-11-CLA may affect peripheral immune cells, thereby promoting anti-inflammation in the brain, since peripheral immune cells such as macrophages or T-cells are also associated with neuroinflammation^33,34^.

Recently, knockout of the IL-10 gene in mice has been found to promote Aβ clearance^35^, while IL-10-overexpression mice have increased Aβ deposits^36^, suggesting an adverse role of IL-10 in amyloid clearance. These results disagree with our results showing an Aβ decrease by *c*-9, *t*-11-CLA feeding, which is accompanied by the upregulation of IL-10. In this regard, we assume that the decrease in Aβ levels by *c*-9, *t*-11-CLA feeding may occur independently from IL-10 expression, while IL-10 expression is involved in the beneficial effects on anti-inflammation. Alternatively, since the previous studies are based on IL-10 gene-knockout mice^35^ or IL-10-overexpressing mice^36^, the conditions are unusual in that IL-10 is knocked out even in cells where a certain amount of IL-10 expression is needed, or there is constant overexpression of IL-10 in all cells. We assume that the effects of IL-10 under physiological conditions, where its expression is only induced in the right cells and within optimal levels, may be different from those in IL-10-deficient mice or mice overexpressing IL-10. Indeed, our results showed that Aβ levels are reduced by an increase in astrocytes expressing IL-10. Further study will be needed to establish the role of IL-10 in AD pathology in *c*-9, *t*-11-CLA diet-fed AD model mice.

We next determined whether dietary *c*-9, *t*-11-CLA enters the brain. A recent study reported that docosahexaenoic acid and also other plasma free fatty acids cross the BBB through Msd2a transporters in the form of LPC^25^. As expected, our results showed that the *sn*-1-*c*-9, *t*-11-CLA-LPC level was increased in the brain of *c*-9, *t*-11-CLA diet-fed mice, including the cortex, hippocampus, cerebellum, brain stem, and olfactory bulb, as well as the liver, whereas no significant change in the LA-LPC level was observed. These results clearly indicated that dietary *c*-9, *t*-11-CLA crosses the BBB, most likely via the Mfsd2a transporter, and enters the brain in the form of *c*-9, *t*-11-CLA incorporated into LPC^25^. Interestingly, the relative abundance of *c*-9, *t*-11-CLA-LPC in the hippocampus or brain stem was much higher than that in the cortex or cerebellum. This may explain the different effects of *c*-9, *t*-11-CLA feeding on Aβ accumulation between the hippocampus and the cortex as mentioned before. At present, whether *sn*-2-*c*-9, *t*-11-CLA-LPC was also increased by the *c*-9, *t*-11-CLA diet is unclear, because the peak position of *sn*-2-*c*-9, *t*-11-CLA-LPC in LC–MS/MS overlapped with that of *sn*-1-LA-LPC, the level of which may be more abundant than that of *sn*-2-*c*-9, *t*-11-CLA-LPC.

In addition, we found that the levels of free *c*-9, *t*-11-CLA and total *c*-9, *t*-11-CLA were significantly increased in *c*-9, *t*-11-CLA diet-fed mouse brains. The level of total *c*-9, *t*-11-CLA was ~ fivefold higher than that of free *c*-9, *t*-11-CLA, suggesting that dietary *c*-9, *t*-11-CLA was incorporated into various lipids including lysophospholipids. Therefore, *c*-9, *t*-11-CLA itself or lipids containing *c*-9, *t*-11-CLA including *c*-9, *t*-11-CLA-LPC or both are likely to be involved in Aβ reduction or the anti-inflammatory response in the brain of *c*-9, *t*-11-CLA diet-fed AD model mice.

Recently, it was reported that oral administration of water containing a nanodroplet of pomegranate seed oil comprising large levels of punicic acid reduces Aβ accumulation and prevents cognitive decline in AD model mice, most likely through *c*-9, *t*-11-CLA, because the administered punicic acid is rapidly metabolized into *c*-9, *t*-11-CLA in the liver, and *c*-9, *t*-11-CLA was also detected in the brain of mice given a nanodroplet of pomegranate seed oil^37^. This study also supports our results showing the beneficial effects of dietary *c*-9, *t*-11-CLA on AD pathogenesis. In addition, the study strongly suggested that *c*-9, *t*-11-CLA confers neuroprotection in an AD mouse model by calpain inhibition^37^. Therefore, dietary *c*-9, *t*-11-CLA likely provides beneficial effects on AD pathogenesis through several pathways, including anti-inflammation, Aβ generation, or Aβ clearance and neuroprotection, possibly by calpain inhibition.

In addition, the dietary intake of butter enriched in *cis*-9, *trans*-11 CLA has been shown to cause memory enhancement through upregulating the expression of phospholipase A2 in rat brain tissue, as assessed by increased latency in the step-down inhibitory avoidance task^38^, and the administration of Nano-PSO has also been reported to prevent cognitive decline in 5XFAD mice^37^. Therefore, it is possible that the dietary intake of *cis*-9, *trans*-11 CLA improves memory performance.

In this study, we showed that dietary *c*-9, *t*-11-CLA reduces Aβ accumulation accompanied by upregulation of anti-inflammatory cytokines in AD model mice. In addition, we clearly demonstrated that intake of dietary *c*-9, *t*-11-CLA leads to an increase in the levels of free *c*-9, *t*-11-CLA and *c*-9, *t*-11-CLA-LPC in the brain.

Thus, intake of dietary *c*-9, *t*-11-CLA will be effective for the prevention of AD progression by decreasing Aβ levels and enhancing anti-inflammatory effects. Further studies on the effects of *c*-9, *t*-11-CLA on memory loss in AD and the biological action of various lipids containing *c*-9, *t*-11-CLA including *c*-9, *t*-11-CLA-LPC will be needed to firmly establish the beneficial effects of prevention of AD progression or inflammation.

## Methods

All methods were carried out in accordance with relevant guidelines and regulations.

### *c*-9,* t*-11-CLA feeding of AD model mice

AD model mice expressing hAPP and bearing the Swedish and Indiana mutations (hAPPSwInd, J20) under the control of the human platelet-derived growth factor beta polypeptide promoter were obtained from The Jackson Laboratory (Bar Harbor, ME, USA). C57/BL6 (wild-type) mice were obtained from Charles River (Yokohama, Japan). hAPPSwInd mice were fed 0.4% *c*-9, *t*-11-CLA (Abcam Inc.), 2.0% sunflower oil (FUJIFILM Wako Pure Chemical Co.), and 97.6% rodent diet CE-2 (CLEA Japan Inc.) from 6 to 14 months old for the *c*-9, *t*-11-CLA diet, and hAPPSwInd mice were fed 2.4% sunflower oil and 97.6% rodent diet CE-2 for the control diet according to the method of a previous report^39^. We provided the necessary amount of food for one week, calculated as an intake of approximately 4 g food/day/mouse every week after confirming the food intake. Mice were sacrificed via carbon dioxide asphyxiation. The left hemisphere of the brain was fixed in 4% buffered paraformaldehyde solution at 4 °C and was cut into 30-μm thick sections using a cryomicrotome for immunohistochemical analysis, as previously reported^40^. Brain regions (cortex, hippocampus) were dissected from the right hemisphere and used for Aβ ELISA.

### Animals

All animal studies were conducted in compliance with the ARRIVE guidelines (approved # P-01-070 by the Ethics Committee for Animal Research of Iwate Medical University). Animals were used under the Guidelines for the Animal Experiments of Iwate Medical University and the Act on Welfare and Management of Animals of Japan. All experimental protocols were evaluated and approved by the Ethics Committee for Animal Research of Iwate Medical University (approval number: P-01-070). Mice were housed in a 12-h light/dark cycle with food and water. We used 6 month-old C57BL/6 J (wild-type) and AD model mice.

### Antibodies

Mouse anti-phosphorylated tau antibody (AT100) was purchased from Thermo Scientific (Carlsbad, CA, USA). Chicken anti-GFAP antibody and goat anti-IBA-1 antibody were purchased from Abcam (Tokyo, Japan). Rabbit anti-IL-10 antibody was purchased from GeneTex (Alton, CA, USA). Rat anti-CD45 antibody was purchased from BioLegend (San Diego, CA, USA). Mouse anti-CD206 antibody was purchased from R&D systems (Minneapolis, MN, USA). Mouse anti-Aβ antibody (6E10) was purchased from BioLegend (San Diego, CA, USA). Rat anti-CD86 was purchased from Thermo Fisher Scientific (Tokyo, Japan).

### Aβ measurement

The cortex and hippocampus were lysed on ice in guanidine hydrochloride buffer (5 M guanidine hydrochloride, 50 mM Tris-HCl, pH 8.8). Aβ40 and Aβ2 in the cortex and hippocampus were measured using a sandwich ELISA kit (Wako, Osaka, Japan). The levels of Aβ40 and Aβ42 were normalized based on the weight of the brain. All samples were measured in duplicate.

### Immunohistochemistry

Immunohistochemistry was performed as previously described^40^. Immunostaining of phosphorylated tau was performed using a Vectastain ABC Kit (Vector Laboratories, Burlingame, CA, USA). For immunofluorescence, brain sections were incubated with primary antibody overnight at 4 °C, and then visualized with Alexa Fluor488- and Alexa Fluor594-tagged secondary antibodies (Life Technologies-Invitrogen, Carlsbad, CA; Abcam). Thioflavin-S staining was performed as previously described^40^. Images of the brain sections were captured under light microscopy or fluorescence microscopy.

### Synthesis of CLA-LPC

Synthesis of CLA-LPC was carried out by NARD Institute, Ltd. Briefly, *sn*-1-CLA-LPC and *sn*-2-CLA-LPC were synthesized by esterification of glycerophosphocholine with *c*-9, *t*-11-CLA prepared as previously reported^41^. The end products were confirmed by ^1^H NMR and LC-MS. The purities of *sn*-1-CLA-LPC and *sn*-2-CLA-LPC were estimated to be approximately 88% and 77%, respectively, by high-performance liquid chromatography analysis.

### LC–MS/MS analysis of brain lipids

For LC–MS/MS analysis, brains from wild-type mice fed *c*-9, *t*-11-CLA (0.4% *c*-9, *t*-11-CLA, 2.0% sunflower oil, and 97.6% rodent diet CE-2) from 6 to 10 months old, and wild-type mice fed the control diet (2.4% sunflower oil, 97.6% rodent diet CE-2) from 6 to 10 months old were used. The brains were frozen in liquid N_2_ and homogenized in methanol (pH 4.0) containing an internal standard of 1 µM 17:0-LPC and 100 nM 17:0-LPA, as previously described^27^. After centrifugation, the supernatants were filtered and analyzed. LC–MS/MS analysis was performed as previously described with some modifications^27^. Briefly, we used an LC–MS/MS system that consisted of a Vanquish UHPLC and TSQ Altis triple quadrupole mass spectrometer (Thermo Fisher Scientific). LC was performed using a reverse phase column [CAPCELL PAK C18 (1.5 mm I.D. × 250 mm, particle size 3 µm)] with a gradient elution of solvent A [5 mM ammonium formate in 95% (v/v) water, pH 4.0] and solvent B [5 mM ammonium formate in 95% (v/v) acetonitrile, pH 4.0] at 150 µl/min. Gradient conditions were as follows: hold 50% B for 0.5 min, followed by a linear gradient to 100% B over 18 min; hold 100% B for 7 min; return to the initial condition over 0.5 min; and maintain for 3 min until the end of the run. LPC was detected with multiple reactive monitoring in positive mode.

### Gas chromatographic analysis of brain lipids

Distilled water (1 ml) was added to 250 mg frozen brain tissue and homogenized until uniform. Lipids were extracted from the samples using 5 ml chloroform/methanol/1.5% KCl (2:2:1, by volume) according to the Bligh-Dyer method. The solvent was removed from the sample using a rotary evaporator. For free fatty acid analysis, 1 mg extracted lipid was methylated with 2 ml methanol, 3 ml benzene, and 0.15 ml 1% trimethylsilyl diazomethane and incubated at room temperature for 30 min. For total fatty acid analysis, 1 mg extracted lipid was first saponified with 0.2 ml 0.5 M KOH in methanol at 100 °C for 10 min. The sample was then methylated with 2 ml 4% methanolic HCl and 1 ml dichloromethane and incubated at 50 °C for 20 min. *n*-heptadecanoic acid (0.01 mg) was used as the internal standard. After methylation, the fatty acid methyl esters were quantitatively determined with a Shimadzu GC-2010 gas chromatograph equipped with a flame-ionization detector and a split-less injection system and fitted with a capillary column (TC-70; 60 m long × 0.25 mm i.d.; GL Sciences Inc., Tokyo, Japan). Fatty acids were identified by comparing retention times to known standards. The amount of CLA was calculated using the internal standard.

### Statistical analyses

Statistical analyses were conducted using t-tests. *P* < 0.05 was considered statistically significant.

### Ethics approval and consent to participate

These experiments were conducted under the guidelines and supervision of the Ethics Committee of Iwate Medical University (approval number: P-01-070).

## Supplementary Information

Supplementary Information 1.
