# Supplementary material for: Dietary cis-9, trans-11-conjugated linoleic acid reduces amyloid β-protein accumulation and upregulates anti-inflammatory cytokines in an Alzheimer’s disease mouse model
Source: Sci Rep. 2021 May 12;11:9749. doi: 10.1038/s41598-021-88870-9 (PMC8115273; doi:10.1038/s41598-021-88870-9)
Supplement: Supplementary file 1 — Supplementary Information 1. [file 41598_2021_88870_MOESM1_ESM.docx]

**Change list**

Page1. The title has been changed as follows.

“with the upregulation of “ has been changed to “regulates“

That’ all.

**Supplementary file**

**Dietary** ***cis*-9, *trans*-11-conjugated linoleic acid reduces amyloid β-protein accumulation and upregulates anti-inflammatory cytokines in an Alzheimer’s disease mouse model**

Yu Fujita, Kuniyuki Kano, Shigenobu Kishino, Toshihiro Nagao, Xuefeng Shen, Chiharu Sato, Hatsune Hatakeyama, Yume Ota, Sho Niibori, Ayako Nomura, Kota Kikuchi, Wataru Yasuno, Sho Takatori, Kazunori Kikuchi, Yoshitake Sano, Taisuke Tomita, Toshiharu Suzuki, Junken Aoki, Kun Zou, Shunji Natori and Hiroto Komano

**Supplemental figure legend**

**Supplemental figure 1**

**CD45+ cells are increased in the hippocampus of AD model mice**

Immunostaining of brain sections of wild-type and AD model mice with anti-CD45 antibody. CD45 (green)-positive cells were only observed in the brain sections of AD model mice.

**Supplemental figure 2**

**The** ***c*-9, *t*-11-CLA diet upregulates the number of IL-19-expressing astrocytes in the hippocampus of AD model mice**

(A) Immunostaining of brain sections of wild-type and AD model mice with anti-IL-19 antibody. (B) Immunostaining of *c*-9, *t*-11-CLA diet-fed and control diet-fed AD model mice with anti-IL-10 antibody. IL-19 (green)-positive cells were significantly increased in the hippocampus of *c*-9, *t*-11-CLA diet-fed AD model mice compared with controls (lower panels). IL-19 (green)-positive cells were not observed in the cortex (upper panels). (C) Double immunostaining of brain sections in the hippocampus of *c*-9, *t*-11-CLA diet-fed and control diet-fed AD model mice with anti-GFAP (astrocyte marker), anti-IBA-1 (microglia marker), and anti-IL-19 antibodies. A portion of GFAP (green)-positive cells were IL-19 (red) positive as shown by yellow color (left merge panels), but IBA-1 (green)-positive cells were not co-localized with IL-19 (red)-positive cells (right merge panels). Bar, 20 µm. WT: wild-type mice, AD: AD model mice.

CLA+: *c*-9, *t*-11-CLA diet-fed AD model mice, CLA−: control diet-fed AD model mice.
